# Hypoglycemia Induced by Zinc Supplementation for COVID-19 Prophylaxis: A Case Series

**DOI:** 10.7759/cureus.38828

**Published:** 2023-05-10

**Authors:** Alissa I Elanjian, Adam Elder, Ribhi Hazin

**Affiliations:** 1 Department of Medical Education, Michigan State University College of Human Medicine, East Lansing, USA; 2 Department of Medical Education, Wayne State University School of Medicine, Detroit, USA; 3 Department of Internal Medicine, Wayne State University School of Medicine, Detroit, USA

**Keywords:** complementary and alternative medicine (cam), mineral supplements, vitamin d, vitamin c, zinc, covid-19 prophylaxis, covid-19, blood glucose, hypoglycemia

## Abstract

There has been significant research and therapeutic activity within the healthcare sector in response to the coronavirus disease 2019 (COVID-19). In the United States, a complementary and alternative medicine (CAM) treatment regimen for improving patients’ immune systems against COVID-19 prophylaxis includes excess zinc, vitamin C, and vitamin D supplementation administered over a seven-day period. Despite the fact that zinc and other mineral supplements are becoming increasingly popular in Western culture, clinical research on CAM remains limited. This case series examines three patients treated with a surplus of zinc tablets for COVID-19 prophylaxis who presented with moderate-to-severe hypoglycemia. Varying amounts of glucose were administered to these patients to offset their low blood sugar levels. Medical staff noted a positive Whipple’s triad in two of the patients but observed no other abnormalities in the laboratory values. All three patients were instructed to cease zinc tablet intake upon discharge. Our findings raise awareness of the potential dangers associated with mineral supplements and serve as a warning for those seeking CAM treatment options.

## Introduction

The global spread of coronavirus disease 2019 (COVID-19) has prompted extensive research on severe acute respiratory syndrome coronavirus 2 (SARS-CoV-2). As understood, the virus binds to human alveolar cells and stimulates both the innate and adaptive immune systems. Clinical studies have shown that patients with acute COVID-19 symptoms have high levels of pro-inflammatory cytokines and impaired immune function [1]. These effects trigger cytokine release and overstimulation eventually causing cytokine release syndrome, an inflammatory reaction that further induces acute respiratory distress syndrome (ARDS).

Researchers have experimented with various COVID-19 treatments. Because the virus leads to inflammation and immune system dysregulation, nutraceuticals have been studied as potential anti-inflammatory and immunomodulatory therapies [2]. Alternatively, a 2022 narrative review examined the potential of complementary and alternative medicine (CAM) for treating COVID-19. The review discussed the relationship between zinc, vitamin C, and vitamin D micronutrients and severe COVID-19 ailments. Results revealed a significant correlation between deficient zinc levels and increased virus complications. There appears to be controversy surrounding the effects of vitamin C and vitamin D levels. However, deficiencies of these essential biochemical cofactors are regarded as risk factors for viral infectivity [1].

Physicians in the United States have been integrating CAM practices by prescribing relatively high doses of zinc, vitamin C, and vitamin D to enhance patients’ immune systems against COVID-19. The patients in this series followed variations of the following treatment protocol over a seven-day period: 250 mg of zinc, 1,000 mg of vitamin C, and 1,000 units of vitamin D. Even with the potential advantages of mineral supplements, limited research exists on the potential for micronutrients to cause or exacerbate human health conditions [3].

This article explores a potential link between micronutrients, particularly zinc, and hypoglycemia. Hypoglycemia is a physiological state characterized by low blood sugar levels. Although its effects may not manifest until plasma glucose concentrations fall below 55 mg/dL, a concentration below 70 mg/dL is typically enough to warrant a diagnosis [4]. Following the intake of excessive zinc tablets to treat COVID-19 symptoms, three patients were diagnosed with moderate-to-severe hypoglycemia and treated accordingly.

## Case presentation

Case one

A 43-year-old male with a body mass index (BMI) of 29 kg/m^2^ and no significant past medical history experienced his first hypoglycemia episode at a local gym. He was found collapsed on the ground by Emergency Medical Services (EMS) with a blood glucose level of 44 mg/dL. He was working out when he reported feeling nauseous and weak while on the elliptical. He attempted to slow down the machine before collapsing. As a result of his fall, the patient suffered a contusion to his left elbow. EMS administered 1 amp of glucose on site and transported the patient to the emergency room. Upon arrival at the Emergency Department (ED), a fingerstick point-of-care test revealed a blood glucose level of 80 mg/dL. Clinicians administered juice and another amp of glucose to normalize his condition.

The patient was diagnosed with COVID-19 three weeks before the collapse, though the possibility of false test results should be noted. The patient reported following an immune-boosting treatment protocol of 1,000 units of vitamin D per day, 1,000 mg of vitamin C per day, and 250 mg of zinc per day for seven days. However, he continued taking over-the-counter zinc tablets as a precautionary measure for more than two weeks after his diagnosis.

An electrocardiogram (EKG) and chest X-ray (CXR) were performed, revealing no abnormalities independent of the stage, lung injury severity, and other disease complications associated with COVID-19. Practitioners also conducted a complete blood count (CBC), hemoglobin A1C (HbA1c), comprehensive metabolic panel (CMP), and thyroid-stimulating hormone (TSH) test. A summary of the results can be found in Table 1.

**Table 1:** Summary of laboratory findings associated with symptoms of hypoglycemia. WBC: white blood cells; Hb: hemoglobin; HCT: hematocrit; HbA1c: hemoglobin A1C; BUN: blood urea nitrogen; TSH: thyroid-stimulating hormone

Parameters	Case one	Case two	Case three	Reference range
WBC (bil/L)	9.2	6.2	7.2	4.5–11.0
Hb (g/dL)	14.9	15.3	15.9	12.0–15.0
Hct (%)	48	54	57	35.0–49.0
Platelet count (µL)	324,000	366,000	322,000	150,000–450,000
HbA1c (%)	4.7	5.0	5.1	<5.7
Creatine (mg/dL)	0.9	1.01	0.7	0.6–1.30
Sodium (mmol/L)	141	139	144	136–145
Potassium (mmol/L)	4.4	4.6	3.9	3.5–5.1
Chloride (mmol/L)	98	99	99	98–107
Carbon dioxide (mEq/L)	24	27	30	21–31
BUN (mg/dL)	8	10	11	7–18
TSH (mIU/L)	1.3	2.5	2.1	0.5–5.0

The patient was observed for 12 hours following testing and discharged home with instructions to avoid supplements.

Case two

A 34-year-old Hispanic male, with a past medical history of hypertension and currently taking 2.5 mg per day of amlodipine, collapsed at his primary care physician’s office. In response to a blood glucose reading of 26 mg/dL, staff administered glucose and called 911. The patient was obtunded with a glucose level of 52 mg/dL when EMS arrived. He received more glucose in the field from EMS and presented to the ED with a glucose level of 38 mg/dL. He was then given dextrose 5% in water (D5W).

Five days before collapsing, the patient was diagnosed with COVID-19, though there is a possibility of misdiagnosis with every testing method. The patient had begun consuming zinc supplements and reported taking one to three 50 mg tablets per day depending on the severity of his symptoms. Before his doctor’s appointment, the patient admitted to ingesting three zinc tablets (150 mg total) an hour earlier.

EKG and CXR tests ordered for the patient were normal for the stage, lung injury severity, and other disease complications associated with COVID-19. A summary of the CBC, HbA1c, CMP, and TSH tests is presented in Table 1. A positive Whipple’s triad was noted upon examination, which was evidenced by the patient’s symptoms of an adrenergic response, a confirmed low blood glucose level at the time of his symptoms, and rapid resolution of the symptoms after the hypoglycemia was corrected. The finding of Whipple’s triad allowed physicians to exclude other neurologic causes of hypoglycemia.

After three hours of D5W and a two-hour waiting period, the patient was discharged with a blood sugar level of 108 mg/dL. He was instructed to follow up with his primary care provider and did not report any further hypoglycemic episodes.

Case three

A 29-year-old male with a past medical history of asthma collapsed at work while using a computer. EMS was called and measured his blood sugar at 40 mg/dL. The patient was transported to the ED and received 3 amps of glucose, which increased his blood sugar to 98 mg/dL after two hours.

Two days before his collapse, the patient visited an urgent care center where he was diagnosed with COVID-19. The possibility of false test results should be noted. He was recommended to take 1,000 mg of vitamin C per day and 250 mg of zinc per day, but he received no indication of when treatment should be discontinued.

EKG and CXR tests revealed no abnormalities relative to the stage, lung injury severity, and other disease complications associated with COVID-19. Pertinent results of the CBC, HbA1c, CMP, and TSH tests are presented in Table 1. A positive Whipple’s triad was observed, as indicated by the patient’s manifestation of an adrenergic response, a low blood glucose level during the onset of symptoms, and rapid relief of symptoms after the correction of hypoglycemia.

The patient was monitored for two more hours before being discharged home to follow up with his primary care provider. No episodes of hypoglycemia or HbA1c fluctuations have been reported since the incident.

## Discussion

It is widely understood that glucose is the brain’s primary source of metabolic fuel. As such, regulatory measures have evolved in humans to sustain adequate serum glucose levels. Gluconeogenesis and glycogenolysis are two biochemical processes that generate glucose from non-carbohydrate sources. Low insulin levels, resulting from low glucose levels, trigger these measures. Acute hypoglycemia regulations include the secretion of glucagon and epinephrine. In prolonged hypoglycemic states, cortisol and growth hormone are released in the body [4]. These hormones trigger the breakdown of fat for energy in a process known as lipolysis. During lipolysis, triacylglycerols are split into glycerol and free fatty acids. The glycerol is used to fuel gluconeogenesis, while the fatty acids are converted into ketone bodies that manifest in the blood. High plasma ketone body concentrations indicate a significant depletion of carbohydrates [5].

Several interventions can be employed to treat hypoglycemia. D5W combined with glucose infusion is the primary protocol for severe symptoms. Conscious patients may consume carbohydrate sources or receive glucagon injections. Education and lifestyle dietary modifications are effective non-pharmacological remedies for patients with recurrent hypoglycemia episodes [1].

According to current understanding, the most common cause of hypoglycemia is diabetes mellitus. Hypoglycemic states have also been associated with fasting or exogenous sources, including alcohol, hormone deficiencies, and pharmacological interventions [1]. Although an unclear relationship exists between most mineral supplements and low blood sugar symptoms, zinc capsules have gained popularity in diabetes research.

Zinc is involved in various physiological functions. As a micronutrient, it contributes significantly to human metabolism, growth, and development. Zinc’s metallic cofactor properties aid in the function of proteins necessary for cell proliferation, differentiation, and death. By suppressing the creation of reactive oxygen species and providing oxidative stress protection, zinc contains powerful antioxidant properties [6].

The impact of zinc on insulin secretion and glucose metabolism is of particular interest to this article. Zinc is highly concentrated in the pancreas, playing a significant role in islet cell functioning and insulin action. Proper regulation of zinc levels is highly dependent on transporter ZnT8, which transports zinc into beta cells of the pancreatic islets. This process is known to help generate, store, and secrete insulin under natural biological conditions. Under pathological conditions, impaired ZnT8 function is believed to impair insulin action when glucose is present, which advances the development of type 2 diabetes and insulin resistance [6,7]. Studies have detected enhanced insulin sensitivity with zinc supplementation in animal models experiencing insulin resistance [7], but more research is warranted with human subjects [6].

Zinc is also thought to impact carbohydrate metabolism by increasing the activity of important enzymes involved in glycolysis and gluconeogenesis. Research indicates that zinc reduces glucose absorption in the intestines while decreasing glucose synthesis in the liver. Concurrently, zinc promotes the breakdown of available glucose and speeds up glycogen storage [7]. Using zinc supplementation interventions in type 2 diabetic patients, a meta-analysis found clinically relevant decreases in fasting blood glucose levels [6]. Two meta-analyses further revealed that diabetic patients who took zinc supplementation for their condition had reduced fasting blood glucose levels, two-hour postprandial glucose levels, and HbA1c levels [7]. Another meta-analysis sought to determine if zinc supplements correlated with risk factors for type 2 diabetes or cardiovascular disease. A combination of low zinc supplement doses (<25 mg/dL) for a long duration (≥12 weeks) improved the greatest number of risk factors for both diseases in individuals, suggesting that zinc has potential benefits even outside of diabetes care [8].

The functions of zinc in insulin secretion and carbohydrate metabolism are believed to collectively enhance glycemic control. It is critical to note that research on zinc and the potential benefits of supplementation links baseline levels of zinc intake with adequate blood glucose maintenance. Some translational studies point to hypoglycemic effects induced by zinc supplementation as an outcome of improved insulin sensitivity [6].

Further research is necessary to debunk the potential adverse effects of zinc. This article takes the first step by presenting three patients with low blood sugar levels amid no other current health issues besides a recent COVID-19 diagnosis and excessive consumption of zinc tablets. It is especially worth mentioning that the patients were not diabetics and did not have any previous history of diabetes. Given zinc’s reasonably established role in lowering the blood sugar levels of diabetic patients, impaired glycemic control is a probable consequence of zinc overdose, especially for individuals without diabetes. While we cannot establish direct causality, both previous research and our results appear to support an association between high doses of zinc intake and hypoglycemia.

Our case report findings also have implications for medical practice revisions. Greater patient education is needed to prevent zinc tablet overconsumption and the potential health risks they pose. Incorporating safer and better-understood medications into COVID-19 treatment protocols may also benefit patients’ health. Both of these points call for continued research into the realm of mineral supplements and CAM therapies.

## Conclusions

This article describes the experiences of three patients who developed moderate-to-severe hypoglycemia after taking excessive zinc tablets to treat COVID-19 symptoms. The case studies and current research provide valuable insights into how mineral supplements may induce medical conditions. Although the effects of CAM on the body are not fully understood, patients run the risk of misinformation, misuse, and health complications. Future research is necessary to deepen our understanding of zinc-induced hypoglycemia and how CAM interventions function within the broader field of human health.

## References

[1] Shakoor H, Feehan J, Al Dhaheri AS (2021). Immune-boosting role of vitamins D, C, E, zinc, selenium and omega-3 fatty acids: could they help against COVID-19?. Maturitas.

[2] Pedrosa LF, Barros AN, Leite-Lais L (2022). Nutritional risk of vitamin D, vitamin C, zinc, and selenium deficiency on risk and clinical outcomes of COVID-19: a narrative review. Clin Nutr ESPEN.

[3] Firouzi S, Pahlavani N, Navashenaq JG, Clayton ZS, Beigmohammadi MT, Malekahmadi M (2022). The effect of vitamin C and Zn supplementation on the immune system and clinical outcomes in COVID-19 patients. Clin Nutr Open Sci.

[4] Mathew P, Thoppil D (2023). Hypoglycemia. https://pubmed.ncbi.nlm.nih.gov/30521262/.

[5] Kolb H, Kempf K, Röhling M, Lenzen-Schulte M, Schloot NC, Martin S (2021). Ketone bodies: from enemy to friend and guardian angel. BMC Med.

[6] Barman S, Srinivasan K (2022). Diabetes and zinc dyshomeostasis: can zinc supplementation mitigate diabetic complications?. Crit Rev Food Sci Nutr.

[7] Ranasinghe P, Pigera S, Galappatthy P, Katulanda P, Constantine GR (2015). Zinc and diabetes mellitus: understanding molecular mechanisms and clinical implications. Daru.

[8] Pompano LM, Boy E (2021). Effects of dose and duration of zinc interventions on risk factors for type 2 diabetes and cardiovascular disease: a systematic review and meta-analysis. Adv Nutr.

